# Early-life infection dynamics and genomic diversity of adenoviruses in a wild primate (Theropithecus gelada)

**DOI:** 10.1099/mgen.0.001595

**Published:** 2025-12-09

**Authors:** Maya J. Saroff, Abebaw Azanaw Haile, Alice Baniel, Simona Kraberger, Melanie Regney, Balázs Harrach, Győző L Kaján, Amy Lu, Jacinta C. Beehner, Thore J. Bergman, Noah Snyder-Mackler, Arvind Varsani, India A. Schneider-Crease

**Affiliations:** 1 School of Life Sciences, Arizona State University, Tempe, AZ, USA; 2 Ethiopian Wildlife Conservation Authority, Debark, Ethiopia; 3 Institut des Sciences de l'Évolution de Montpellier UMR5554, CNRS, IRD, EPHE, Université de Montpellier, Montpellier, France; 4 The Biodesign Center of Fundamental and Applied Microbiomics, Arizona State University, Tempe, AZ, USA; 5 HUN-REN Veterinary Medical Research Institute, Budapest, 1143, Hungary; 6 Department of Anthropology, Stony Brook University, Stony Brook, New York, USA; 7 Department of Anthropology, University of Michigan, Ann Arbor, MI, USA; 8 Department of Psychology, University of Michigan, Ann Arbor, MI, USA; 9 Department of Ecology and Evolution, University of Michigan, Ann Arbor, MI, USA; 10 Center for Evolution and Medicine, Arizona State University, Tempe, AZ, USA; 11 School of Human Evolution and Social Change, Arizona State University, Tempe, AZ, USA; 12 Structural Biology Research Unit, Department of Integrative Biomedical Sciences, University of Cape Town, Cape Town, 7925, South Africa; 13 School of Technology for Public Health, Arizona State University, Phoenix, Arizona, USA

**Keywords:** *Adenoviridae*, geladas, infancy, metagenomics, primate virome, *Theropithecus gelada*

## Abstract

In humans, adenoviruses (AdVs) are frequently associated with respiratory illnesses, posing risks to children with developing immune systems and immunocompromised individuals. Outbreaks and epidemics are generally centred in close-contact settings, such as childcare facilities, and transmission occurs through faecal–oral and airborne pathways. AdVs have coevolved across the primate lineage, but very little is known about whether the early-life dynamics in non-human primates mirror those in humans. Here, we leverage longitudinal data collected on a population of geladas (*Theropithecus gelada*) in the Simien Mountains National Park, Ethiopia, to evaluate AdV dynamics across the gelada lifespan. We identified ten coding-complete AdV genomes representing seven unique simian adenovirus (SAdV) types, four of which are adequately different from the known ones to establish new species. We assessed behavioural and seasonal drivers of SAdV presence and richness across repeated faecal samples from known individuals. Contrary to our expectation that the highest risk would occur after the initiation of play behaviour in infancy (~6 months of age), when peer-to-peer transmission risk is expected to increase, SAdV likelihood was highest in infants under 6 months of age. Risk and richness declined over the lifespan, with very few adults infected, and higher minimum temperatures were weakly but significantly negatively associated with richness. Our results suggest that, unlike in humans, SAdV exposure occurs prior to the initiation of close-contact play behaviours and likely results from the close spatial proximity of conspecifics throughout the dependent period. Like AdVs in humans, SAdVs in geladas maintain low levels in adulthood, with early infections potentially conferring life-long immunity.

**Impact Statement**

While adenoviruses often cause mild illnesses, they can develop into more pathogenic forms or recombine with other strains to pose public health risks. Understanding adenoviruses in wild primates – which share close genetic and ecological ties with humans – advances our understanding of viral evolution, zoonotic potential, transmission dynamics and ecosystem health. Our assessment of adenovirus dynamics over time in geladas (*Theropithecus gelada*) offers a rare opportunity to identify how these viruses operate in natural contexts. This underlines the utility of the One Health framework; by leveraging evolutionary and ecological perspectives to examine viral dynamics, we can better understand and manage viruses of public health importance.

## Data Summary

All genomes have been deposited into GenBank (accession nos. PQ490708–PQ490717). All other code and data are available at https://github.com/GeladaResearchProject/Saroff_et-al_adenovirus. All raw reads are deposited under BioProject no. PRJNA795171, BioSamples nos. SAMN43383109–SAMN43383118 and SRA nos. SRR30416927–SRR30416936.

## Introduction

Adenoviruses (AdVs) (family *Adenoviridae*) can cause diseases in the respiratory, gastrointestinal, urinary and ocular systems of their vertebrate hosts, including humans [1,3]. These non-enveloped dsDNA viruses have ~24–48 kbp genomes that include 26–721 bp inverted terminal repeats [4] and are classified into six genera (*Aviadenovirus*, *Barthadenovirus*, *Ichtadenovirus*, *Mastadenovirus*, *Siadenovirus* and *Testadenovirus* [5,6]). Humans and other primates are infected with AdVs in the genus *Mastadenovirus*, which are transmitted through faecal–oral or airborne (e.g. aerosol and droplet) routes [2,7, 8] and can cause conjunctivitis, gastroenteritis, pneumonia and the common cold. Homologous recombination, in which genomes exchange genetic segments and increase viral variation, facilitates efficient viral replication and the emergence of new variants with pathogenic potential [3,9].

Disease severity and transmissibility vary across serotypes and interact with host immunocompetence [3,10, 11]. In humans, the highest severity is found among children and the immunocompromised, with broad tissue tropism among these viruses reflected in the variety of their pathogenic manifestations [12,13]. Long-lasting cellular and humoral immunity from infections contributes to lower observed incidence in adults, although latent AdVs can reactivate later in life with changes in immune status [3,11, 14]. Outbreaks and epidemics tend to be centred in close-proximity settings such as military camps and childcare facilities [1,2, 15]. Despite the attention to AdV dynamics across human populations, and while AdVs within the same genus have been identified across our closest primate relatives, very little is known about the dynamics of AdVs outside of the human context.

The coevolution of AdVs with their primate hosts is reflected in the identification of AdVs across the primate lineage [16,17]. AdVs have been found in wild and captive apes [18,19], Central and South American (platyrrhines) and Afro-Asian (catarrhines) primates [18,24] and lemurs (strepsirrhines) (17, 16). Most studies in primates offer cross-sectional or case study perspectives that are useful in identifying host breadth and describing general patterns, but are generally constrained to collecting samples from adults, which limits our understanding of the generalizability of early life AdV patterns.

Here, we leverage longitudinal data collected on a wild population of geladas (*Theropithecus gelada*), graminivorous primates endemic to the Ethiopian Highlands, to assess AdV dynamics across the lifespan using repeated faecal sampling from known individuals. We identified seven adenovirus genomes in our study population – four types meriting the establishment of novel species and three types belonging to previously established species – and leveraged repeated sampling of infants to assess the impact of developmental, social and seasonal variables on AdVs in geladas. Infants begin exploring their surroundings and playing with other infants from their own social group and from other closely associated groups after ~6 months of age [25], transition from maternal milk to a graminivorous diet at ~18 months [26] and become independent from their mothers at about 2.5 years [27]. Reductions in grass availability during the dry season increase feeding on underground resources that are more time-consuming to extract and are linked to lower rates of play [28], which could lower AdV exposure during the hot, dry season. If the dynamics of AdVs in the wild mirror those of AdVs in humans, we would thus expect AdV risk to be highest in individuals after 6 months of age (when playing starts), among infants in closely associated social groups, and during the colder, wetter months.

## Methods

### Sample collection

Faecal samples were collected from known geladas between October 2015 and March 2020 in the Simien Mountains National Park, Ethiopia, immediately following observed defecation and were stored at room temperature in RNAlater. Samples were taken from the middle of the faecal bolus to prevent contamination. This study population has been under long-term observation by the Simien Mountains Gelada Research Project since 2006. Researchers collect near-daily demographic, behavioural and life history data on individuals in this population and can identify individuals with high accuracy. Geladas live in complex, multi-level societies in which multiple ‘reproductive units’ of related adult females, their offspring, a tenure-limited dominant ‘leader male’ and one or more subordinate ‘follower males’ preferentially associate to form loose ‘bands’ [29]. These bands, composed of multiple reproductive units, are generally – but not always – found together. For this study, we collected 248 faecal samples from 43 individuals across 14 units in 2 bands. We collected samples from dependent infants unlikely to yet be exploring their environment or playing (<6 months), infants still dependent on their mothers but more likely to spend time exploring and playing (~6 months–2.5 years), independent juveniles to young adults (~2.5–5 years) and adults (>5 years, Table 1). This included repeated sampling of infants across these categories. All infants (*n*=21) were sampled in more than one category (median=2 category changes). We collected rainfall and temperature data throughout the study period.

**Table 1.** Sample distribution across demographic categories

	<6 months		6 months–2.5 years		2.5–5 years		>5 years		Total	
	Samples	Individuals	Samples	Individuals	Samples	Individuals	Samples	Individuals	Samples	Individuals
**Female**	16	10	62	10	13	8	40	11	131	21
**Male**	17	9	67	11	21	9	12	11	117	22
**Total**	33	19	129	21	34	17	52	22	248	43

### Sample library preparation and sequencing

We used the QIAGEN DNeasy PowerLyzer PowerSoil DNA Isolation Kit (cat. no. 12855) to process faecal samples with modifications described in [30]. Faecal crude lysates, obtained following lysis, bead-beating and inhibitor removal, were used for downstream metagenomic library preparation using a modified version of the Illumina DNA Prep Kit (cat. no. 20060059) protocol (https://doi.org/10.17504/protocols.io.rm7vzbob4vx1/v1). For each sample, we diluted 2 µl of crude lysate input to 20 µl with nuclease-free water and performed tagmentation with a reduced volume of 5 µl each of kit buffers (BLT and TB1). We ran 12 cycles of PCR with reduced reaction volumes (10 µl of EPM PCR mix, 2.5 µl of each 10 µM primer per sample). We then completed library preparation according to the manufacturer’s instructions and sequenced libraries on an Illumina Novaseq 6000 S4 flow cell (2×150 bp).

### Metagenome assemblies and adenovirus identification

We used Cutadapt to trim adapters and low-quality bases from the raw reads and removed reads mapping to the gelada genome using the Burrows–Wheeler Alignment Tool [31]. We used MetaSPAdes [32] to *de novo* assemble the resulting non-host reads into contiguous sequences (‘contigs’). Contigs >1000 nt were analysed against a RefSeq (version 220) viral protein database using diamond blast [33] with an *e*-value threshold of 10^−5^. Adenovirus-like contigs were extracted and analysed using Cenote-Taker3 for viral genome identification and annotation [34], and the annotations were manually checked and reannotated for coding regions with introns based on adenovirus gene functional studies.

### Analyses of the adenoviruses

A curated dataset of representative adenovirus species (one per species, across known genera) was downloaded from GenBank. The DNA polymerase coding region was extracted from these, as well as those from this study. These were translated and aligned using MAFFT v7 [35]. The resulting alignment was used to infer a maximum likelihood phylogenetic tree using PhyML 3.0 [36] with the LG+I+G substitution model as determined by ProtTest 3 [37] with aLRT branch support. Branches with <0.8 aLRT support were collapsed with TreeGraph 2 [38].

Intergenomic identities were determined using VIRIDIC [39]. All genome-wide and amino acid sequence pairwise identities were determined using SDT v1.2 [40]. We used BBmap v35.85 [41] to map the raw reads from all the samples to identify the presence/absence (0/1) of the seven AdV lineages of adenoviruses identified in this study. Positive infection was assigned for samples to which reads with greater than 98% identity mapped across more than 10% of each adenovirus genome.

### Statistical analysis

To assess the predictors of the presence of any adenovirus (0/1) in a sample, we fit a generalized linear mixed model (GLMM) with the logit link function using the ‘glmmTMB’ package with R Statistical Software (v4.4.2; Posit Team, 2025). We included individual sex and developmental category (operationally defined as <6 months, 6 months–2.5 years, 2.5–5 years and >5 years) at the time of sampling, average minimum temperature over the 30 days prior to sampling and cumulative rainfall over the 90 days prior to sample collection (scaled) to account for potential seasonal and dietary sources of variation [30], band (i.e. broad social group) membership and total number of non-host sample reads (log-transformed). Individual ID was added as a random effect. To assess the predictors of adenovirus richness (i.e. the number of unique adenovirus types per sample), we fit another GLMM using ‘glmmTMB’ with the same fixed and random effects structure, using a Poisson distribution and log link function to account for count data.

## Results

### Adenoviridae genomes

**Fig. 1.**
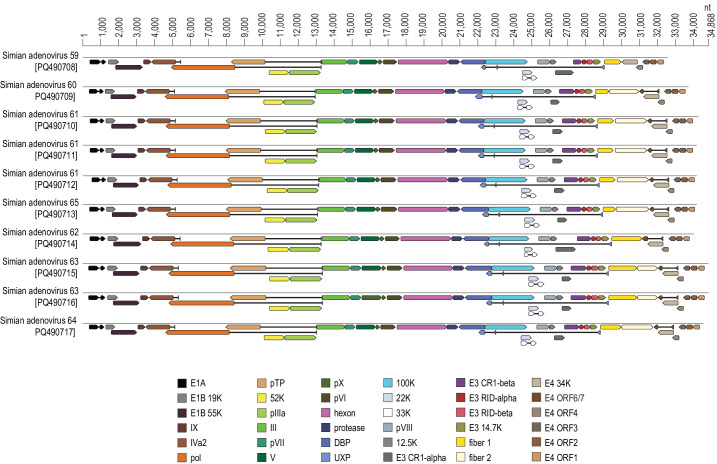
Genome organization of the ten genomes identified from the *de novo* assemblies that represent seven unique AdV lineages (genus: *Mastadenovirus*).

From the *de novo* assemblies, we identified ten complete coding AdV genomes in the *Mastadenovirus* genus. These ten genomes (32,612–34,868 nt in length) represent seven unique lineages, which we named simian adenovirus (SAdV)-59, -60, -61, -62, -63, -64 and -65, and have been deposited in GenBank under the accession numbers PQ490708–PQ490717. An illustration of the genome organizations and the coding regions of these ten SAdVs is provided in Fig. 1.

**Fig. 2.**
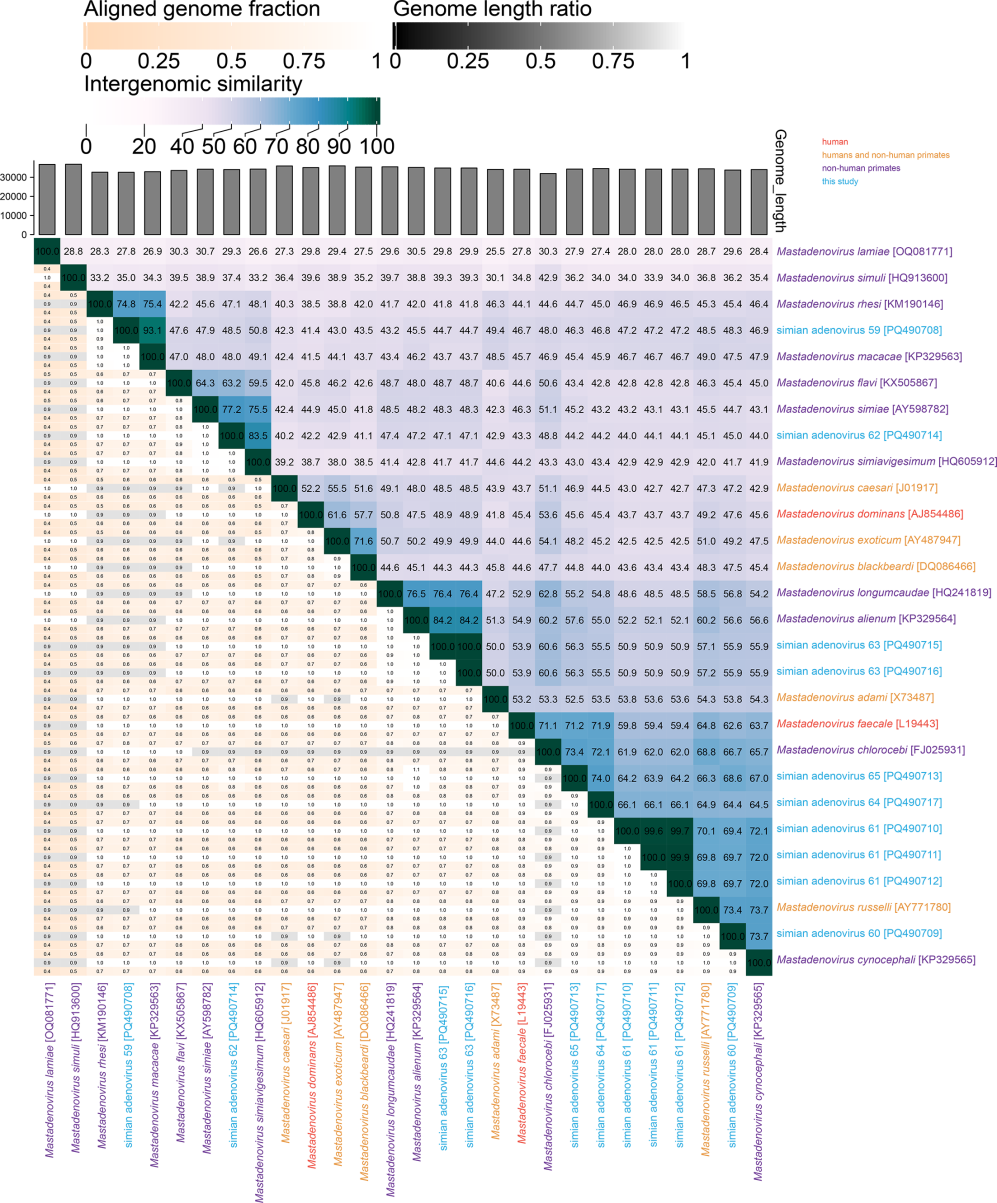
Intergenomic pairwise identities of the ten AdVs (genus: *Mastadenovirus*) identified in this study with representative primate AdVs that have been classified into species.

The intergenomic pairwise identities determined by VIRIDIC [39] are summarized in Fig. 2. The highest measured sequence identities are as follows. Simian adenovirus 59 (PQ490708) shares 93.1% intergenomic identity with simian adenovirus 13 (KP329563), classified in the species *Mastadenovirus macacae* isolated from a macaque and later identified in yellow baboons [16,24]. SAdV-60 (PQ490709) and SAdV-61 (PQ490710–PQ490712) share 72.0–73.7% pairwise identity with SAdV-19 (KP329565; *Mastadenovirus cynocephali*) in yellow baboons (*Papio cynocephalus*) [24]. The SAdV-62 (PQ490714) genome is most closely related to that of SAdV-20 (HQ605912) in the species *Mastadenovirus simiavigesimum* [18] found in grivets. The two SAdV-63 (PQ490715, PQ490716) genomes share 84.2% pairwise identity with that of SAdV-16 (KP329564), classified in *Mastadenovirus alienum* and found in a grivet (*Chlorocebus aethiops*) [16,24]. SAdV-64 (PQ490717) and SAdV-65 (PQ490713) share 74% intergenomic similarity between them and 72.1–73.4% with SAdV-18 (FJ025931), classified in the species *Mastadenovirus chlorocebi* and found also in a grivet monkey (*C. aethiops*) [18]. Furthermore, the two SAdV-63 (PQ490715 and PQ490716) genomes share 99.9% pairwise identity, and the three genomes of SAdV-61 (PQ490710–PQ490712) share >99.6 % identity.

**Fig. 3.**
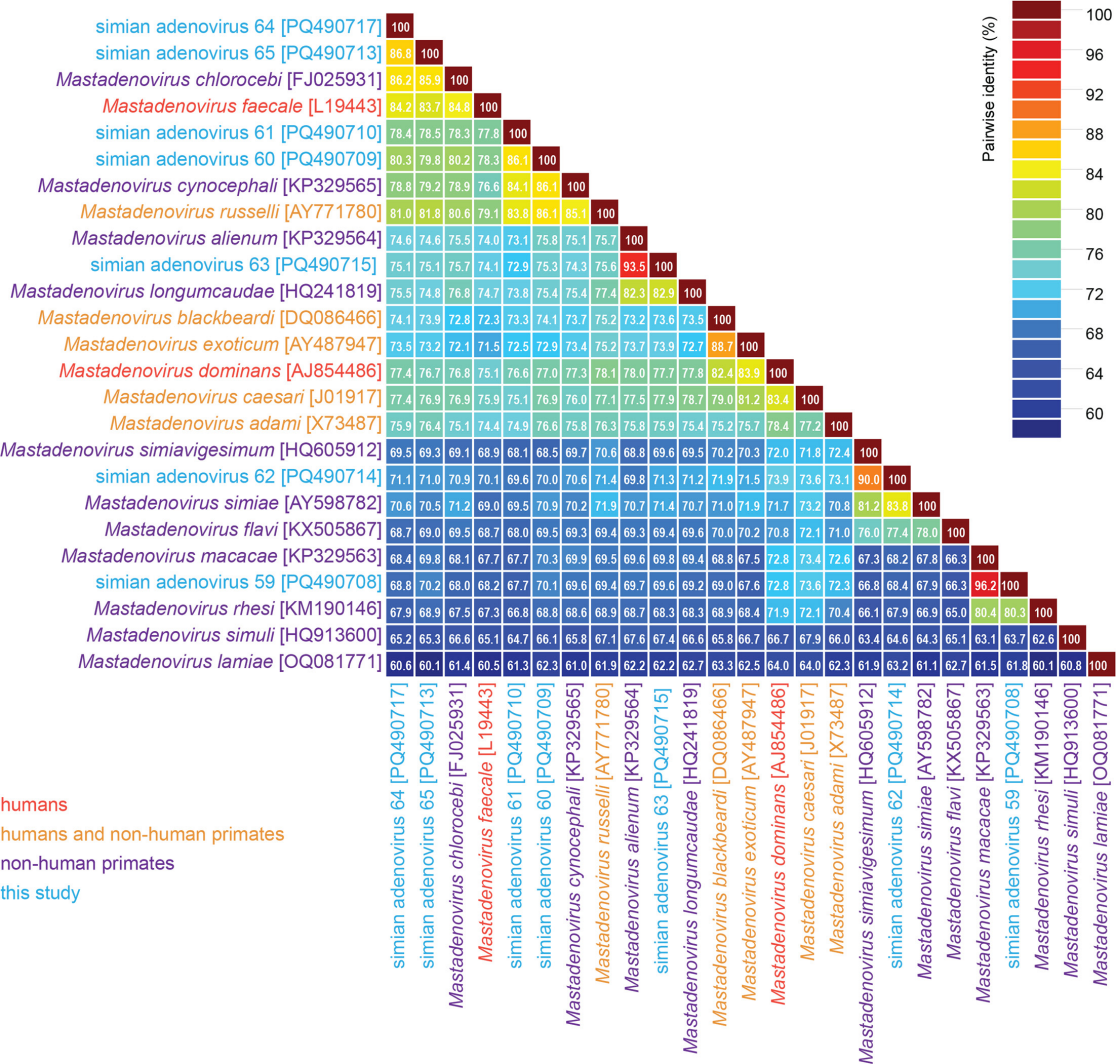
Pairwise identity matrix of the DNA polymerase amino acid sequences of representative members of each of the species of primate-associated AdVs. Phylogenetic relationships of AdVs are constructed with DNA polymerase because it is highly conserved across all AdVs.

**Fig. 4.**
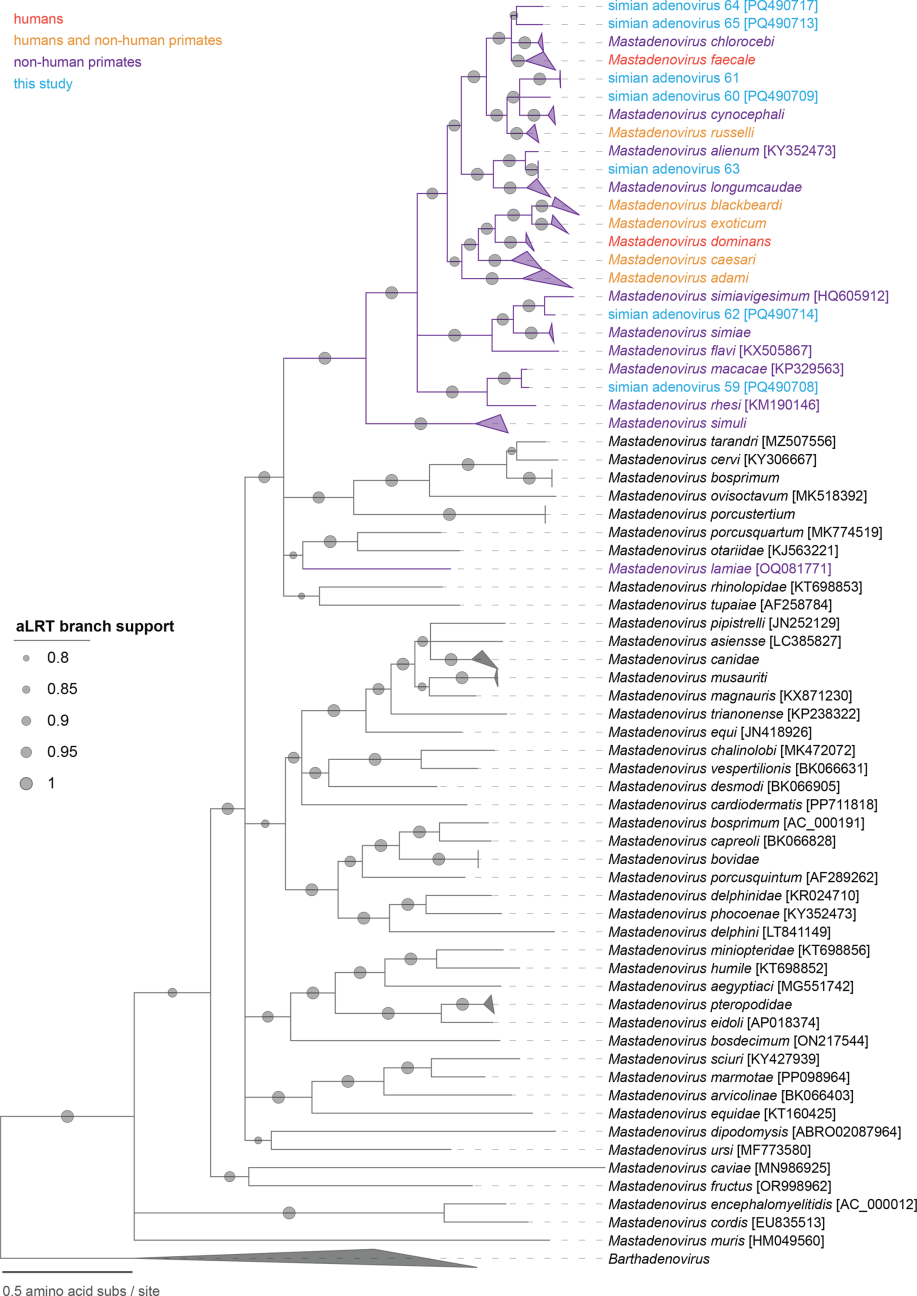
Maximum likelihood phylogenetic tree of representative DNA polymerase amino acid sequences of members of the *Mastadenovirus* genus with the AdVs identified in this study.

Members of the family *Adenoviridae* are classified into species mainly based on the phylogenetic distance of the DNA polymerase amino acid sequences [5]. A >15 % pairwise identity difference to the closest member of an accepted species usually merits the establishment of a new species, but the phylogeny of all AdVs (monophyletic clustering) and other demarcation criteria may also be important: host, GC percentage and genome organization (e.g. number of fibre genes). Thus, if the DNA polymerase amino acid sequences share <85–90 % pairwise identity with those of classified adenoviruses, they can be considered to represent new species. Of the seven lineages of mastadenoviruses from geladas identified in this study, SAdV-59 (PQ490708), SAdV-62 (PQ490714) and SAdV-63 (PQ490715 and PQ490716) are classified in an established species (*Mastadenovirus macacae*, *Mastadenovirus simiavigesimum* and *Mastadenovirus alienum*, respectively). The DNA polymerase amino acid sequences of SAdV-59 and SAdV-13 (KP329563; *Mastadenovirus macacae*) share 96.2%, SAdV-62 and SAdV-20 (HQ605912, *Mastadenovirus simiavigesimum*) share 90.0% and SAdV-63 and SAdV-16 (KP329564; *Mastadenovirus alienum*) share 93.5% pairwise identity (Fig. 3). SAdV-60, -61, -64 and -65 all represent members of four new species in the genus *Mastadenovirus* as their DNA polymerase sequences share lower percentage amino acid identity (60–86 %) with those of other classified members and with each other (Fig. 3). This is also supported by the maximum likelihood phylogenetic tree of the DNA polymerase amino acid sequences (Fig. 4).

## Distribution of adenoviruses

### Descriptive statistics

**Fig. 5.**
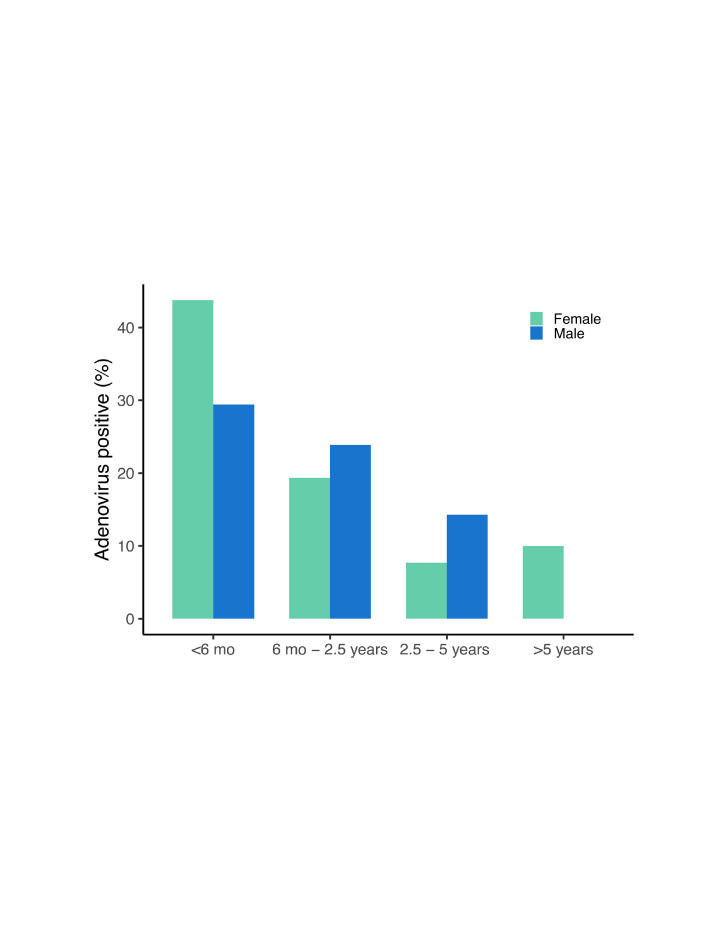
Percentage of faecal samples positive for any adenovirus across developmental categories: under 6 months (*n*=12), between 6 months and 2.5 years (*n*=28), between 2.5 and 5 years (*n*=4) and over 5 years (*n*=4).

At least one of the seven SAdV types (SAdV-59, -60, -61, -62, -63, -64 or -65) was found in 48 of 248 total samples (19.4%) from 22 individuals. SAdV prevalence ranged across developmental categories (Fig. 5): 36.4% of 33 samples from individuals under 6 months of age (12 positive samples: 7 females, 5 males), 21.7% of 129 samples from individuals between 6 months and 2.5 years (28 positive samples: 12 females, 16 males), 11.8% of 34 samples from individuals between 2.5 and 5 years (4 positive samples: 1 females, 3 males) and 8% of 44 samples from individuals over 5 years (4 positive samples: 4 females, no males). Forty-one samples had one SAdV type (85.42%: 41 of 48), six samples had two types (12.5%: 6 of 48) and only one sample had three types (2.08%: 1 of 48). All samples with more than one SAdV type were from individuals under 2.5 years of age.

SAdV-63 had the highest prevalence and SAdV-59 had the lowest (Table 2). All SAdVs (-59, -60, -61, -62, -63, -64 and -65) were found in samples from non-adult individuals, while only three were identified in samples from adults (-59, -60 and -64).

**Table 2.** Distribution of each SAdV among positive samples (*n*=48) and total samples (*n*=248) Asterisks (*) indicate SAdVs closely related to previously described simian adenoviruses.

SAdV	# positive samples	% of positive samples (*n*=48)	% of total samples (*n*=248)
SAdV-59*	7	14.58	2.82
SAdV-60	14	29.17	5.65
SAdV-61	9	18.75	3.63
SAdV-62*	4	8.33	1.61
SAdV-63*	15	31.25	6.05
SAdV-64	6	12.5	2.42
SAdV-65	1	2.05	0.4

### Age predicts adenovirus

**Fig. 6.**
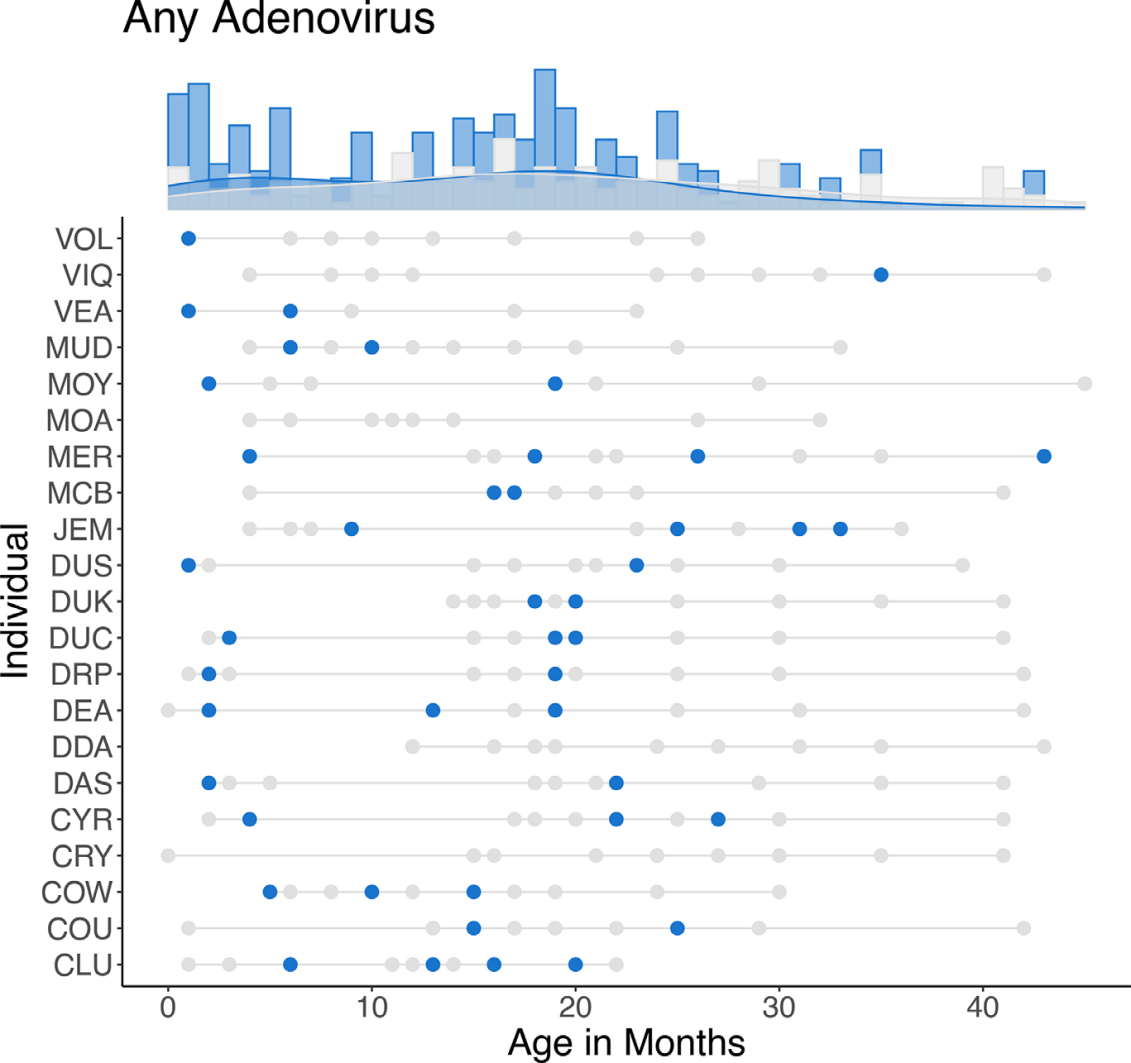
Distribution of samples positive (blue dots) or negative (grey dots) for any SAdV across individuals (rows connected by grey lines) over their infancy. Density trends of positive and negative samples are shown above, with each bar representing the number of samples per age bin scaled independently within each group (positive, negative).

Infants under 6 months of age were significantly more likely to test positive for SAdVs than any other age group (Table S1, Supplementary Material 1): these individuals had ~3 times higher odds of testing positive than 6 months–2.5 years (confidence interval (CI) 1.2, 7.9; *P*=0.019), ~7.8 times higher odds compared to individuals 2.5–5 years (CI 1.9, 32.4; *P*=0.004) and ~9.6 times higher odds compared to individuals over 5 years of age (CI 2.5, 36.7; *P*<0.001, Fig. 6). Infants under 6 months of age also had significantly higher SAdV richness (Table S2 and Fig. S1, Supplementary Material 1) than individuals between 6 months and 2.5 years (*P*=0.013), between 2.5 and 5 years (*P*<0.003) and over 5 years (*P*<0.001). Increasing temperature was weakly but significantly associated with lower SAdV richness (*P*=0.044, Fig. S2, Supplementary Material 1).

## Discussion

We identified seven unique adenovirus lineages (types) in wild geladas, with SAdV-60, -61, -64 and -65 representing new species and SAdV-59, -62 and -63 clustering with and similar to already described SAdV species identified in other closely related – but geographically widespread – primates (grivets, macaques and yellow baboons). Because geladas are relatively isolated, with a range restricted to the highlands of Ethiopia above ~2,000 m [42], the newly described species may be gelada specialists. By contrast, the already described SAdVs, highly similar to those identified in grivets, macaques and yellow baboons, may represent generalists capable of infecting multiple cercopithecoid primates, although host breadth data are currently limited and adenoviruses typically exhibit a narrow host range, infecting only a limited number of closely related species [43]. Additional investigations into the landscape of AdVs in natural environments and across primates will allow for a deeper assessment of adenovirus host range and dynamics.

The vast majority of SAdVs were carried by individuals under 2.5 years of age, which likely reflects the convergence of increased susceptibility stemming from the naive infant immune system [13] and exposure due to close contact with other infants [44,45], as it does in humans. However, contrary to our expectations, the highest SAdV risk was observed in infants under 6 months of age. At ~6 months, gelada infants begin exploring their environments and playing with other infants [25], which involves close-contact behaviours including touching, hair-pulling, soft biting and play fighting [46] that may facilitate AdV transmission via close contact or droplets. Increased risk at ~6 months would thus mirror the childcare facility effect on AdV risk in humans; however, our results suggest that peak SAdV infection precedes the onset of direct peer-to-peer contact. This may be because dependent infants are exposed to other individuals through their mothers’ social interactions (e.g. grooming) and through the close spatial proximity of individuals in reproductive units. Cross-unit play may explain why band membership did not significantly predict SAdV presence or richness; because ‘bands’ are somewhat fluid [29], and infants play outside of their natal units [25], they may disseminate SAdVs across units and between bands.

Early exposure to SAdVs could thus provide early protection for infants by the time they begin playing that may last throughout the lifespan, with the few cases observed in adult females potentially arising from reactivation of latent infections during periods of immune suppression [3,10]. While adult female geladas – particularly those of lower rank – may experience potentially immunosuppressive levels of social stress because of higher aggression over limited resources in the dry season [47,48], we observed a weak negative association between higher minimum temperature and SAdV richness across all samples and no adults with more than one SAdV type at a time. In line with our original hypothesis that predicted higher SAdV rates in colder, wetter months, this likely reflects seasonally reduced opportunities for social contact in younger individuals [28] rather than the increased stress of dry seasons. Moreover, no adult males were identified with any SAdV, suggesting that there may be sex-based differences in susceptibility or exposure. However, low overall sampling of adults constrains our ability to fully assess the nature of these relationships. Expanded sampling of adults over seasons, paired with analyses of stress hormones and behaviour, will be critical to understanding how seasonally driven behavioural changes may shape SAdV dynamics in the wild.

Altogether, our results suggest that early life changes in close contact are the primary drivers of AdV dynamics in the wild, as they are in humans, but that exposure in infants occurs even prior to the initiation of play behaviour in geladas. As in humans, this early exposure to AdVs may confer lasting immunity, with low levels of breakthrough infections later in life. While the low levels of observed SAdVs in adults could be due to easier detection in lower diversity infant microbial communities, further sampling of adults will help clarify how SAdVs affect hosts throughout the lifespan. More broadly, our work adds to the limited understanding of viral diversity and dynamics in both wild [30,42] and captive geladas [49].

## Supplementary material

10.1099/mgen.0.001595 Supplementary Material 1.

## Abbreviations

AdVs: adenoviruses
GLMM: generalized linear mixed model
SAdV: simian adenovirus
