# Supplementary material for: Early-life infection dynamics and genomic diversity of adenoviruses in a wild primate (Theropithecus gelada)
Source: Microb Genom. 2025 Dec 9;11(12):001595. doi: 10.1099/mgen.0.001595 (PMC12688034; doi:10.1099/mgen.0.001595)
Supplement: Supplementary Material 1. [file mgen-11-01595-s001.pdf]

Supplementary Materials

	Estimate	StdError	z value	p value	OR	Confidence Interval (OR)	IOR	Confidence Interval (IOR)
(Intercept)	-4.181	4.806	-0.87	0.384	0.015	0-188.407	65.416	0.005-806236.995
sex (male)	-0.017	0.342	-0.051	0.96	0.983	0.503-1.922	1.017	0.52-1.99
>5 years	-2.264	0.683	-3.316	<0.001	0.104	0.027-0.396	9.62	2.524-36.667
2.5 - 5 years	-2.065	0.722	-2.861	0.004	0.127	0.031-0.522	7.883	1.916-32.429
6 mo - 2.5 years	-1.125	0.48	-2.343	0.019	0.325	0.127-0.832	3.08	1.202-7.891
Rainfall (90 day cumulative, scaled)	0.075	0.175	0.431	0.666	1.078	0.765-1.52	0.927	0.658-1.307
Minimum temperature (30 days)	-0.29	0.156	-1.857	0.063	0.748	0.551-1.016	1.337	0.984-1.817
Total read count (log-transformed)	0.394	0.297	1.324	0.185	1.482	0.828-2.655	0.675	0.377-1.208
social group ("small band")	0.08	0.356	0.226	0.821	1.084	0.54-2.176	0.923	0.46-1.853

Table S1: GLMM model results for predictors of AdV likelihood. This model was fitted with a binomial logit link function. Operational development categories were defined as <6 months (reference category), 6 months–2.5 years, 2.5 years–5 years, and >5 years. Odds ratios and inverse odds ratios are presented with their respective confidence intervals.

Predictor	Estimate	StdError	z value	p value
(Intercept)	-4.517	3.778	-1.196	0.232
sex (male)	0.114	0.274	0.416	0.678
>5 years	-1.952	0.582	-3.352	<0.001
2.5 - 5 years	-1.81	0.6	-3.019	0.003
6 mo - 2.5 years	-0.853	0.343	-2.49	0.013
Rainfall (90 day cumulative, scaled)	0.02	0.141	0.143	0.887

Minimum temperature (30 days)	-0.246	0.122	-2.011	0.044
Total read count (log-transformed)	0.364	0.234	1.558	0.119
social group ("small band")	0.115	0.288	0.399	0.69

Table S2: GLMM model results for predictors of AdV richness. This model was fitted with a Poisson distribution and a log link function. Operational development categories were defined as <6 months (reference category), 6 months–2.5 years, 2.5 years–5 years, and >5 years.

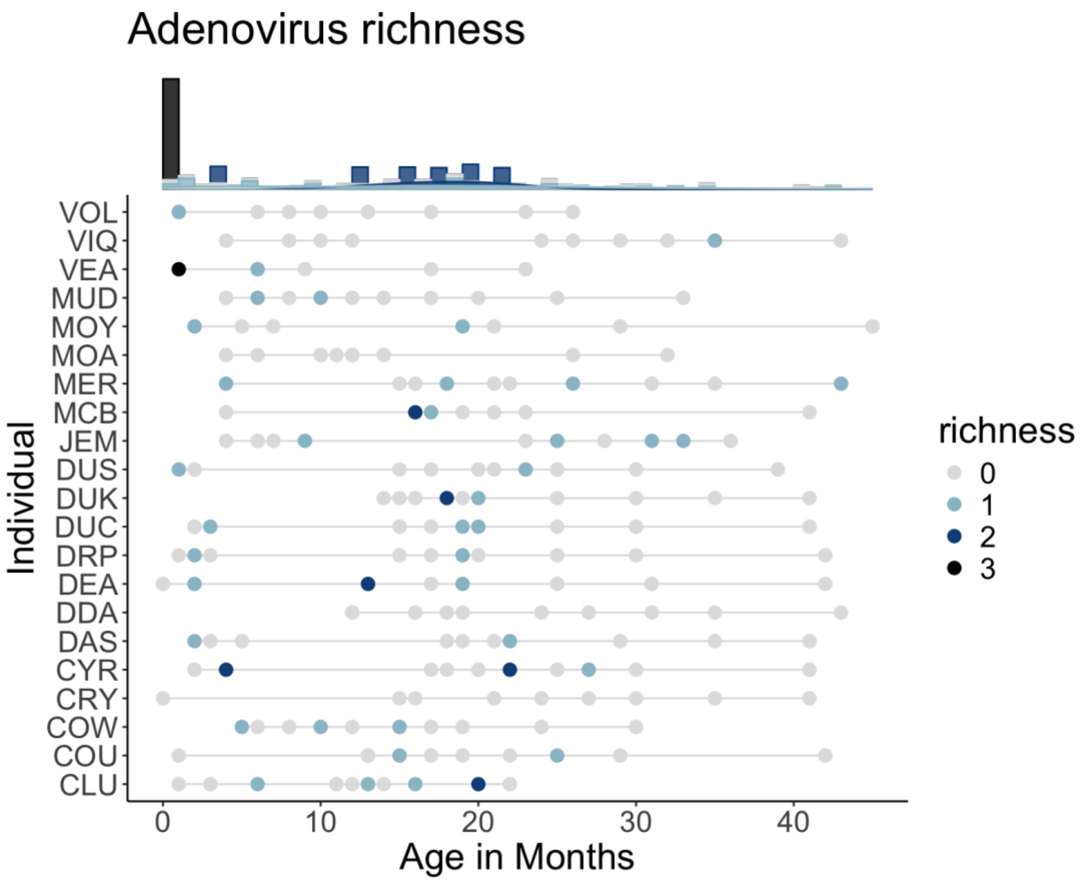

Figure S1: Distribution of adenovirus-positive samples for immature geladas across age (in months). Richness scale (0 to 3) indicates the number of unique adenovirus strains present in a given sample, with grey dots indicating samples with no strains and dark blue indicating samples with higher richness. Horizontal lines indicate sampling (dots) for each individual over time. The histograms on top indicate richness density.

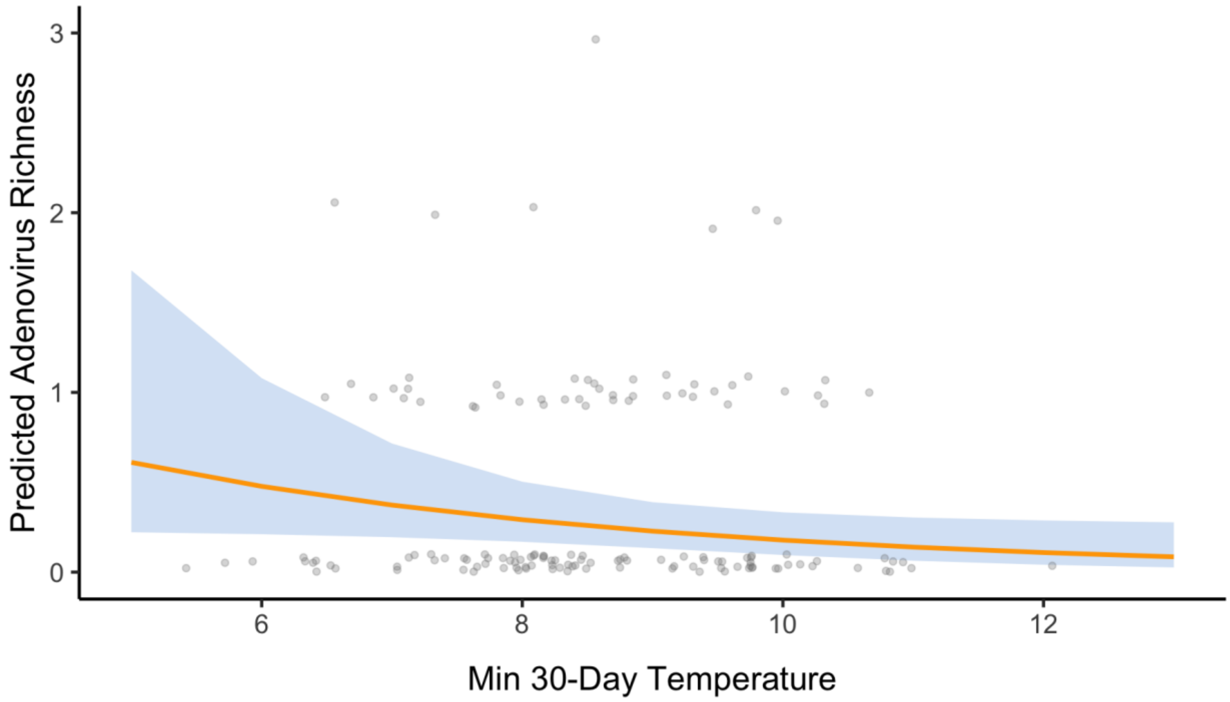

Figure S2: Predicted relationship between minimum temperature across 30 days preceding sample collection and SAdV richness. Predicted SAdV richness (orange line, blue 95% bias-corrected confidence intervals) as a function of temperature estimated from a generalized linear mixed model with a Poisson distribution. Jittered grey points represent observed richness values (0-3) in samples
